# Spatiotemporal Patterns of Adverse Pregnancy Outcomes in Rural Areas of Henan, China

**DOI:** 10.3390/ijerph192315966

**Published:** 2022-11-30

**Authors:** Jian Chai, Junxi Zhang, Yuanyuan Shi, Panpan Sun, Yuhong Wang, Dezhuan Zhou, Wei Dong, Lifang Jiang, Peng Jia

**Affiliations:** 1 National Health Commission Key Laboratory of Birth Defects Prevention, Henan Key Laboratory of Population Defects Prevention, Henan Institute of Reproduction Health Science and Technology, Zhengzhou 450002, China; 2 School of Resource and Environmental Sciences, Wuhan University, Wuhan 430072, China; 3 International Institute of Spatial Lifecourse Health (ISLE), Wuhan University, Wuhan 430072, China

**Keywords:** pre-pregnancy checkup, adverse pregnancy outcome, spatial pattern, spatial analysis, spatial epidemiology

## Abstract

The spatial patterns of adverse pregnancy outcomes (APOs) are complex, vary by place, and remain not entirely clear. This study investigated spatiotemporal patterns of APOs in rural areas of Henan, China. We used data from 1,315,327 singleton pregnancies during 2013–2016 in rural areas of Henan, China, from the National Free Pre-pregnancy Checkup Program (NFPCP). A spatiotemporal analysis of APOs was conducted based on the time of conception and current address. Results of seasonality decomposed showed a slight decline in the incidence rate of APOs (12.93% to 11.27% in the compound trend) among the participants from 2013 to 2016 and also variation in annual periodicity (peaking in autumn at 12.66% and hitting bottom in spring at 11.16%). Spatial clusters of APOs were concentrated in an intersection band of northwestern to southeastern Henan Province (with a relative risk ratio ranging from 3.66 to 1.20), the northwestern and northern portion for temporal variation (having a trend in the cluster ranged from −6.25% to 83.93). This study provides an overall picture of APOs that presented downward trends over time, seasonal fluctuation, and clustered patterns across space and over time in Henan Province—the most populated province in China. The findings of this study warrant future studies to investigate underlying influential factors of spatial variation of APOs.

## 1. Introduction

Adverse pregnancy outcomes (APOs) include low birth weight, fetal macrosomia, preterm delivery, prolonged birth, infant mortality, spontaneous abortion, induced labour, and stillbirth [1,2]. APOs are major health concerns in obstetrics and can affect the health of both mothers and fetuses. According to one cohort study, fetal macrosomia has been associated with severe postpartum hemorrhage, obstetric anal sphincter injury, shoulder dystocia, and obstetric brachial plexus injury [3]. Preterm delivery and low birth weight are still the leading causes of infant morbidity in China according to the data from the national child mortality surveillance system [4]. Preterm delivery has also been linked to a higher incidence of chronic fetal diseases in children, including cardiovascular diseases, cancers, respiratory diseases, and diabetes [5].

Several APOs are continually prevalent. Spontaneous abortion has been regarded as the most common APO, affecting one-third of women [6] and 11–20% of all pregnancies [7]. The estimated global preterm delivery rate was 10.6%, ranging from 9.0% to 12.0% [8]. According to a recent study based on data from the World Bank, premature delivery still ranks first among all causes of under-5 mortality in China, accounting for about 31% of global child mortality [9]. Another Chinese study has found that low birth weight and premature delivery accounted for 13.4% of child mortality based on the national child mortality surveillance system from 2010–2016 [4]. These facts imply that APOs remain a challenge in public health.

Spatial technology has developed rapidly and been used to improve maternal health and predict APOs in recent years [10]. In the state of Ohio in the US, a retrospective cohort study was conducted to understand spatial inequalities in infant mortality and preterm birth preterm delivery, and reported a concentration in urban centers of populations at the highest risk of poor birth outcomes [11]. Similarly, in Ethiopia, a secondary analysis aimed to investigate the spatial distribution and determinants of medical abortion reported that abortion varies across regions [12]. Some studies have also been carried out in China [13]. In Guangdong Province of China, a cohort study has demonstrated regional disparities in the incidence of preterm delivery and low birth weight [14]. The spatial distribution patterns of APOs are complex, vary by place, and remain partially clear [14,15,16]. The spatiotemporal distribution of APOs should be more refined. Although Henan is the largest registered population and the third largest birth population in China, no study has been conducted in this province, which has a distinct economic development level. The issue has grown in importance in light of the spatiotemporal distribution of APOs in Henan Province. We hypothesized that there is a distinct and clustered spatiotemporal distribution of APOs in Henan Province that is of great importance to public health [17,18].

In this study, we examined the spatiotemporal distribution of APOs in Henan Province based on over one million women aged 20–49 extracted from the National Free Pre-Pregnancy Checkup Program (NFPCP) in China. The findings of this study provide an overall picture and a more profound yet refined understanding of APO distribution patterns across space and over time. Thus, it would serve as an essential basis for future APO-related research, such as identifying APO determinants of APOs and targeting and tailoring interventions.

## 2. Materials and Methods

### 2.1. Study Area and Data

**Figure 1:**
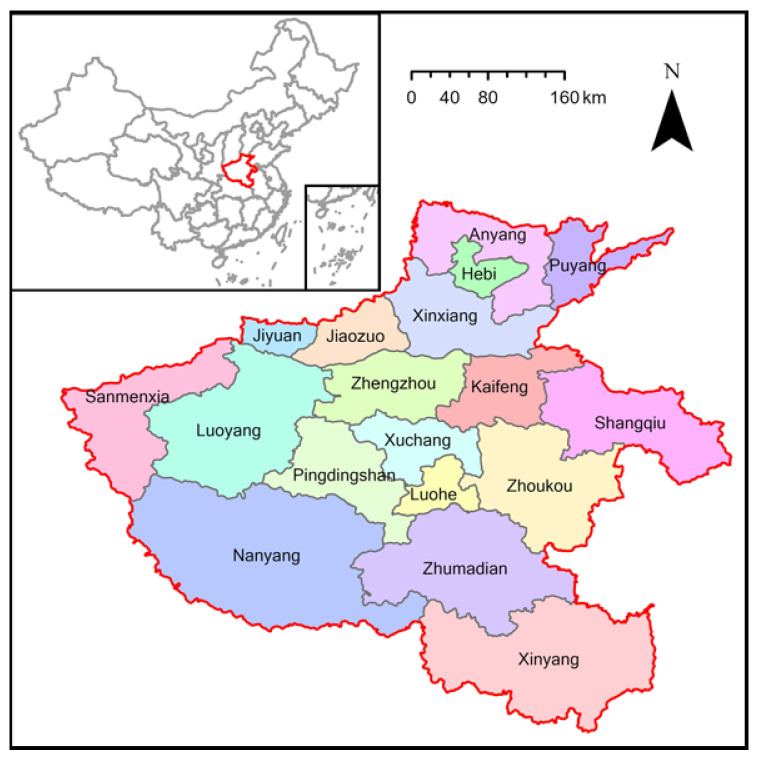
The study area (Henan Province of China).

Henan Province, in central China, has a total population of 104,281,397 as of the 2010 Chinese census and consists of 18 cities, including 50 districts in urban areas and 118 counties in non-urban areas (Figure 1).

This study was conducted based on the National Free Pre-Pregnancy Checkup Program (NFPCP) in Henan, part of a nationwide program in China. Several previous studies have described check-ups and follow-ups for the NFPCP [19,20]. The NFPCP dataset for Henan was collected and maintained anonymously for privacy protection by the Birth Defect Prevention Laboratory of the National Health Committee in the Henan Data Sub-Center of the National Free Pre-Pregnancy Checkup Program. Regarding this cohort study, participants were women with NFPCP from 2013 to 2016 in Henan who reported a pregnancy outcome in 12 months. Baseline characteristics were collected from data from Pre-Pregnancy checkups, including pre-pregnancy physical examination, demographic data, lifestyles, present address, and information on the last menstrual period (LMP). Primary outcomes were from follow-up data. The Henan Institute for Reproductive Health Science and Technology, Henan, China, institutional review board approved this study. All participants provided written informed consent.

In Henan Province, NFPCP was initiated by the National Health Commission and the National Ministry of Finance on 1 January 2010. The target population of this program included all couples preparing to have a pregnancy and having agricultural household registration throughout the province from 2013. It was decided that no fewer than 80% of the target women in Henan Province would benefit from NFPCP by this date. In this study, we originally describe these data as the foundation for the etiological investigation of APOs.

### 2.2. APO Outcomes

Several APOs were ascertained, registered, and regarded as the primary outcomes, including preterm delivery, defined as a live birth before 37 gestational weeks; post-term birth, describing gestational ages beyond term (>42 weeks) [21]; spontaneous abortion as a pregnancy loss of up to 27 weeks and 6 days [22]; low birth weight infant as the delivery with an infant birth weight lower than 2.5 kg [23], and fetal macrosomia as more than 4 kg [3]. Other APOs include medical abortion (≤16 weeks), therapeutic induced labour (between 14 to 27 weeks for a genetic reason) [24], and stillbirth (a fetus born between 20 weeks and 42 weeks of gestation with no evidence of life such as breathing, heartbeat, or movement) [25].

### 2.3. Variables

The maternal age was divided into six groups based on the date of the pregnant woman’s last menstrual period; ethnicity was divided into the Han nationality and others; maternal educational level included “college or higher”, “senior high school”, “junior high school”, and “primary school or below”; occupation was divided into farmers and others. Individuals were classified as underweight (<18.5 kg/m^2^), normal weight (18.5–23.9 kg/m^2^), overweight (24.0–27.9 kg/m^2^), and obese (≥28.0 kg/m^2^) based on their BMI. In NFPCP, cigarette smoking and alcohol drinking for maternal and paternal were all reported as “No”, “Occasionally”, and “Often”. The women’s present addresses were divided into 5 regions according to their geographical location: east, west, middle, south, north (Table 1). The season of conception was defined as the one within which the first day of LMP fell, categorized as spring (March to May), summer (June to August), autumn (September to November) and winter (December to February). The missing values of each variable were recorded as a new category.

### 2.4. Geolocation

Each woman’s address information at the county level when they accepted NFPCP was collected and coded as the area number by the Minister of Civil Affairs of RPC. This information was compared with the hospital’s address where pregnancies were terminated or births given. The cases that show consistency were included and coded. Finally, we joined all cases with geospatial shape file data of Henan Province from a package built-in R named mapchina [26].

### 2.5. Methods of APO Analysis 

The baseline characteristics of the participants were summarized by percentages in the dichotomous categories (whether APOs were present or not). We compared the proportions of categorical variables between the groups using a chi-square test. The Kolmogorov–Smirnov test was used to compare the distributions of ordinal variables between pregnancies with APOs against those without APOs. As the variables with missing data were used only for chi-square tests rather than covariate controlled in statistical models, no sensitivity analysis was conducted for those missing data. Monthly APO incidence rates were decomposed and plotted into seasonal components, combined trend and cycle components, as well as error components [27,28]. The average monthly APO rates at the county/district level were calculated and visualized [26,29,30]. The county/district level of space-time scan analyses were performed to identify spatial clustering patterns of high APO incidence, which have also persisted over time. For pure spatial cluster and spatial variation in trends, the Bernoulli model and discrete Poisson model were employed, respectively. A Monte Carlo hypothesis test based on log likelihood ratio (LLR) was carried out to present all clusters with significant *p* value (*p* < 0.05). The Gini coefficient was used to select the optimal set of clusters to report when secondary spatial clusters were taken into account [31]. Following the methods outlined by Moura [32] and Santos [33], this spatial statistical analysis method involves the development of a circular window that scans the entire study. Finally, a correlation matrix plot was conducted for total and separate incidences of APOs to understand the relationship between different APOs, i.e., to what degree they were spatiotemporally similar. SaTScan (V 9.6) software was automated by using the package rsatscan (v.0.3.9200) for spatial-temporal cluster analysis [34]. All statistical analyses were carried out in R (version 3.6.1).

## 3. Results

### 3.1. Participants

**Figure 2:**
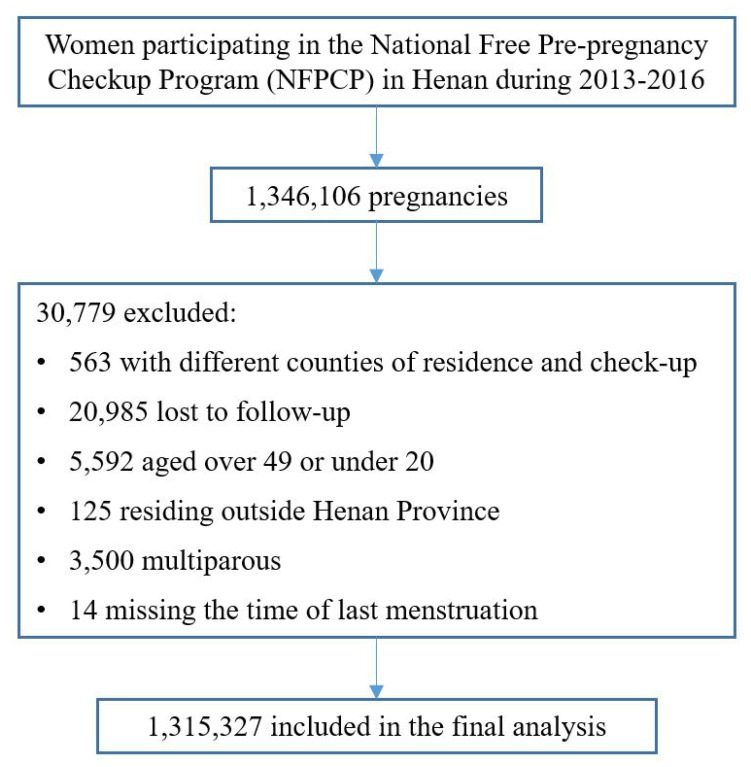
The flowchart of data exclusion and inclusion.

The current study was based on data from NFPCP participants, in Henan from 2013 to 2016, with consequential pregnancies occurring. At the Pre-Pregnancy stage, 1,346,106 participants were recorded as pregnant, with 30,779 participants were excluded and 1,315,327 were included in the final analysis (Figure 2). Additionally, 160,383 participants, approximately 12.19% of the total pregnancies, were recorded as APOs.

### 3.2. Baseline Characteristics by APOs

The demographic characteristics of women with APOs differed from those with term birth. Women with their current residence in North-Eastern Henan showed the highest incidence ratio of APOs, whereas those in the West showed the lowest. As shown in Table 1, autumn pregnancies have the lowest incidence of APOs, while spring pregnancies have the highest, regardless of the temporal distribution.

### 3.3. Temporal Distribution of APOs over Time

**Figure 3:**
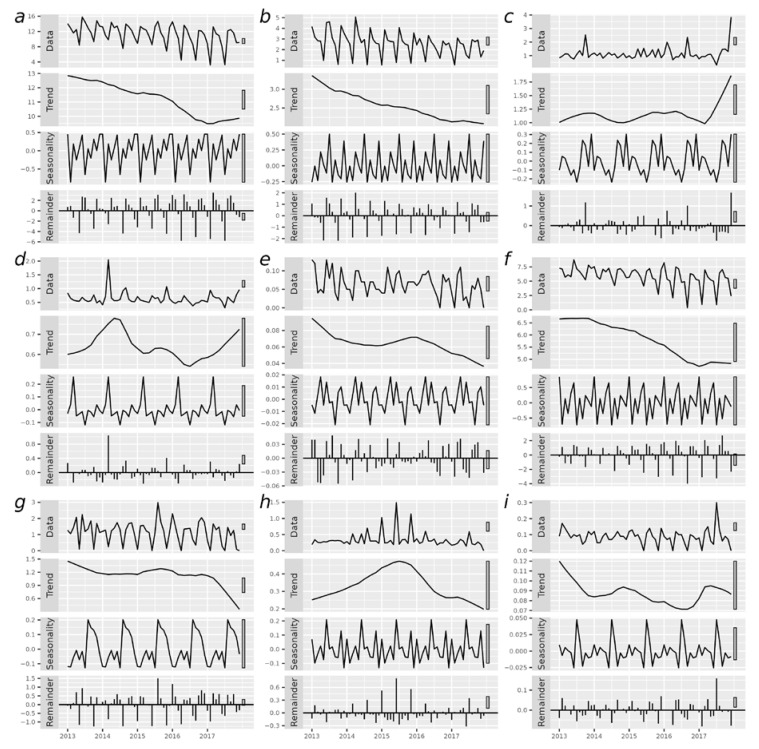
Seasonality decomposition of monthly incidence ratio for all APOs (**a**) and several major APOs separately, including preterm delivery (**b**), spontaneous abortion (**c**), low birth weight infant (**d**), stillbirth (**e**), fetal macrosomia (**f**), prolonged delivery (**g**), medical abortion (**h**), and therapeutic (**i**). The height of the vertical bar on the right of the graph, varying by the range of Y-axis, represents the same value in each panel: 1.31 (**a**), 0.76 (**b**), 0.54 (**c**), 0.23 (**d**), 0.04 (**e**), 1.57 (**f**), 0.32 (**g**), 0.275 (**h**), and 0.0487 (**i**).

The seasonal decomposition plots exhibited the temporal distribution in four aspects: the raw data, trend, seasonal variation, and remainder represent the actual incidence rate of certain diseases, the long-term variation, seasonal variation, and the irregularity of data (Figure 3).

Regarding APOs, a complete result was demonstrated in Figure 3a. High incidence rates of all APOs were observed in a transition period between spring and summer, approximately in May of each year (Friedman test: *p* < 0.001). The graph also showed a steady decline in the incidence of APOs for pregnancies from 2013 to 2016 in rural areas of Henan Province (from 12.93 to 11.27, Cox-Stuart: *p* < 0.001). Based on the remainder, a 12-month stochastic variance was demonstrated (Figure 3a).

Figure 3b–i show the temporal distribution of eight APOs separately. The temporal trends of the 8 APOs were most likely to present a downward trend, except for spontaneous abortion, low birth weight and medical abortion. Spontaneous abortions showed an increasing trend. An increasing trend of LBWI was observed in 2016, whereas medical abortion shows an insignificant trend (Cox-Stuart: *p* > 0.05). The seasonal incidence rate of APOs separately was also presented in Table 2.

### 3.4. Spatial Patterns of APOs

**Figure 4:**
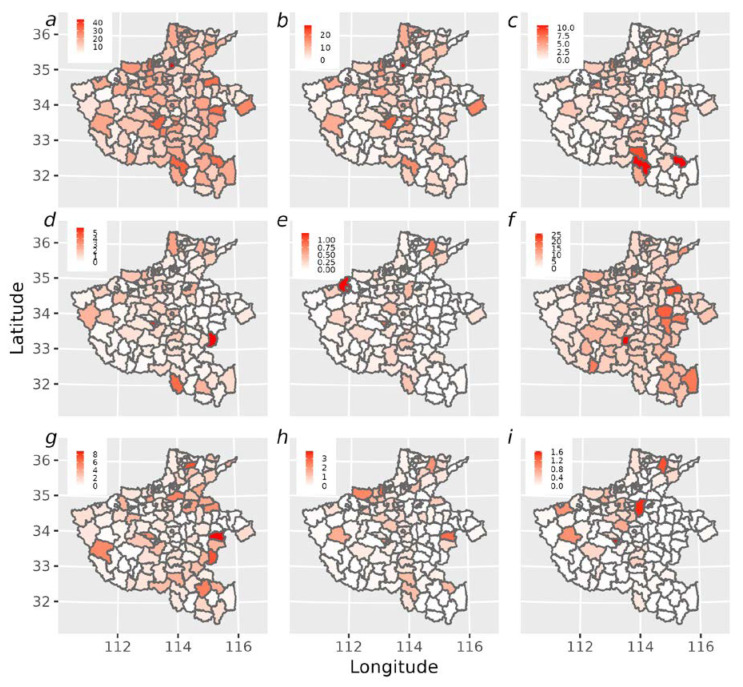
Maps of incidence rates at the county/district level in Henan Province during 2013–2016 for all APOs (**a**), preterm delivery (**b**), spontaneous abortion (**c**), low birth weight (**d**), stillbirth (**e**), fetal macrosomia (**f**), prolonged delivery (**g**), medical abortion (**h**), and therapeutic induction of labour (**i**).

Regarding several significant types of APOs, Figure 4a summarizes the spatial distribution pattern of the incidence rate. Across 158 counties or districts, the incidence of all APOs varied from 0.8% to 41.33% in Henan Province from 2013 to 2016.

The spatial distribution of incidence rates for the eight types of APOs was shown separately (Figure 4b–i). From the data, it can be seen that the significantly high risk emerged consistently in north-central and north-eastern Henan for preterm delivery, spontaneous abortion, low birth weight, medical abortion, and therapeutic inducement of labor. A significantly increased risk of spontaneous abortion also emerged in three counties southeast of Henan, including Queshan County, Pingqiao District, and Huaibin County. Similarly, a higher risk of low birth weight has also emerged in the north-central and two other western counties.

### 3.5. Spatio-Temporal Clustering of APOs

**Figure 5:**
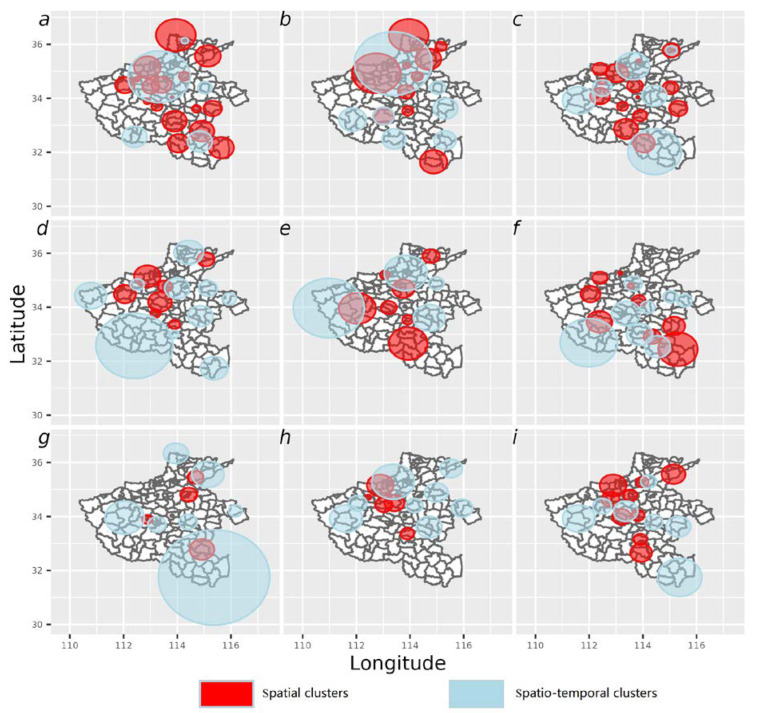
Spatial and spatio-temporal clusters of incidence rates at the county/district level in Henan Province during 2013–2016 for all APOs (**a**), preterm delivery (**b**), spontaneous abortion (**c**), low birth weight (**d**), stillbirth (**e**), fetal macrosomia (**f**), prolonged delivery (**g**), medical abortion (**h**), and therapeutic induction of labour (**i**).

Spatial clusters of higher rates of all APOs is shown in Figure 5. The results showed that statistically significant (*p* < 0.05) spatial clusters of high incidence of APOs were distributed across the north, northwest and southeast of Henan. These areas are filled in red and have a relative risk ratios ranging from 3.66 to 1.20. Of the eight types of APOs separately, clusters with high incidence were consistently concentrated in the northeast for preterm delivery, spontaneous abortion, low birth weight infant, medical abortion and induced labor; clusters of high fetal macrosomia and prolonged delivery were found in the southeast of Henan Province (Figure 5b–i).

The results for spatial-temporal variation in statistically significant clusters with high trends that are evenly distributed across the north, northwest and southeast of Henan filled with light blue clusters ranging from 6.25% to 83.93% (Figure 5a). Of the eight types of APOs separately, clusters with high trends are also consistently concentrated in the northwest for preterm delivery, spontaneous abortion, low birth weight infant, and medical abortion; whereas, clusters with high trends in the Southeast for fetal macrosomia and prolonged delivery (Figure 5b–i).

### 3.6. Spatio-Temporal Correlation across Eight Types of APOs

**Figure 6:**
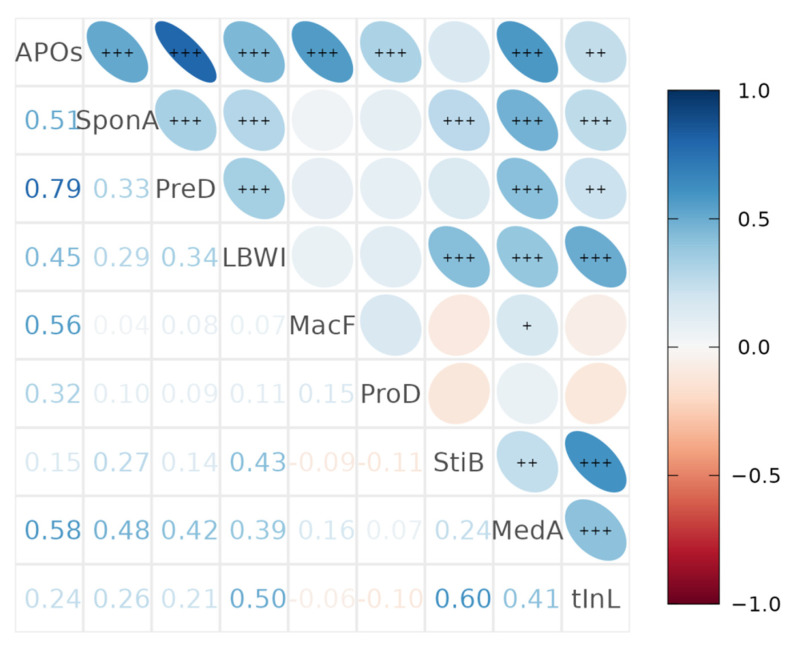
Correlations of monthly incidence rates of APOs at the county/district level in Henan Province during 2013–2016. APOs, all APOs; SponA: spontaneous abortion; PreD: preterm delivery; LBWI: low berth weigh infant; MacF: macrosomia fetus; ProD: prolonged delivery; StiB: stillbirth; MedA: medical abortion; tInL: therapeutic induction of labour. The significant levels are labeled as: +0.05, ++0.01, +++0.001.

The paired Pearson’s correlation coefficient test (two-tailed) was used to assess the relationships of spatiotemporal distribution between the eight types of APOs. For the 36 pairs of APOs, the positive associations ranged from 0.1 to 0.8, *p* < 0.05, with the highest among all APOs–PreD, negative associations ranged from −0.11 to −0.09, *p* < 0.05; when except for all APOs, tInL-StiB and tInL-LBW were the closest two (Figure 6).

## 4. Discussion

In the current study, a large-scale retrospective cohort of 1,315,328 cases of pregnancies between 2013 and 2016, seasonality decomposition analysis, maps of incidence coupled with a spatial-temporal scan statistical method, and Spearman’s correlation analysis were successfully applied to explore spatial and temporal patterns of APOs at the level of the county in Henan Province. The spatiotemporal distribution of APOs was depicted as several disparity patterns, including decreasing trend, seasonal fluctuation, and a high incidence clustered pattern identified, based on the results of our presented study. 

In this study, the monthly incidence rate of all APOs showed a downward trend. In contrast, the eight types of APOs separately or APOs in total at the county level in Henan Province were more complicated and mixed, using seasonality decomposed, after adjusting for seasonal effect, for pregnancies in rural areas from 2013 to 2016. The incidence of all APOs has been declining. Spontaneous abortion and low birth rates showed an upward trend. However, the other six APOs remained consistent and showed downward trends, including preterm delivery, stillbirth, fetal macrosomia, prolonged delivery, medical abortion, and induced labor. This study has been unable to demonstrate an upward trend of APOs identified for preterm delivery by previous research [28], but in line with another study for Shanxi Province, China, low birth weight and preterm delivery showed a downward trend [35]. A possible explanation for this is the downward trend of Cesarean delivery, a preterm delivery risk identified in Henan Province [36]. Another explanation for this was a difference in spatial trends. According to one European study, the rates of singleton preterm delivery are stable or decreased in roughly half of the countries studied, implying that the pregnancy outcome trend may vary by country [37]. As a result, despite presenting a decreasing trend, higher trend clusters with an upward trend were identified in our results using a spatiotemporal cluster scan. These results suggest that the trend in the risks of APOs is likely to vary in space and tempo and challenge a widespread belief that the rising rate of APOs has been the norm. These decreases can be explained by antenatal and pre-conception health care elevation. The National Free Pre-Pregnancy Check-up Program was initiated in 2010. Previous research suggests that women participating in this program will likely increase their risk of certain behaviors, such as smoking and alcohol consumption.

Our results also highlighted the seasonal pattern for APOs. The incidence ratios were consistently lower in total or separately during May and June. These months are a transition period from spring to autumn, which saw the lowest incidence ratios in preterm delivery and MF, except for low birth weight and prolonged delivery. Our results for preterm delivery are corroborated by non-Hispanic white births in North Carolina. These previous findings also suggest that genetic factors can influence seasonal patterns, lending credence to the discrepancy between our results and other researchers’ [38].These results suggested that pregnancy in autumn had the lowest risk of APOs.

A purely spatial cluster scan identified cluster patterns of APOs globally or separately. Interestingly, higher incidence clusters of APOs were contingently identified in north-western Henan Province for preterm delivery, spontaneous abortion, low birth weight, and medical abortion. These results suggest that this district is most likely to be a hot spot for many APOs and has a higher APO risk of incidence. These results confirmed that APOs could be distributed in Henan in cluster patterns aligned with Guangdong [14]. Miao et al. demonstrate a cluster distribution of APOs in Guangdong Province and confirm the association between spatial heterogeneity of APOs with socioeconomic indicators, in line with Charlene C. Nielsen et al. [15]. Furthermore, Yang Zhen et al. classified northern-western Henan as a final-stage district of economic development in 2000 and 2010 using per capita GDP. Based on the theory developed by the last two pieces of research above, socioeconomic heterogeneity might explain the elevated risk of APOs [39]. Air pollution should also be considered in light of our current spatial heterogeneity. Yu Shijie et al. identified differences in the spatial distribution of NO2 between regions higher in the northwest and lower in the southeast [40]. The result in satellite remote sensing data shows a similar spatial pattern of NO2. When comparing these two results with our APOs spatial distribution, we found that the air pollutant was quite likely to be the novelty factors explaining the spatial heterogeneity of APOs. Further work on the association between these two factors and APOs was suggested. Moreover, many factors, including internal and external factors, could contribute to APOs individually or interactively. Most current studies focus on internal factors. For example, Yang et al. [41] explored the effects of maternal iodine status and thyroid diseases on APOs in Henan; Liu et al. found out that higher maternal Pre-Pregnancy weight might increase the risk of APOs [42]. Metzger et al. analyzed the relationship between hyperglycemia and APOs. Environmental factors, on the other hand, should be investigated [43]. In recent years, environmental factors contributing to APOs have garnered increasing attention, such as air pollution and healthy resources. This study investigated the spatial-temporal distribution of APOs as a prerequisite for the further exploration of environmental factors that contribute to APOs. Compared to traditional epidemiology studies, this study used spatial analysis to identify the clusters of high incidences and significant changes. It could provide information for further research into the potential environmental factors of APOs.

Most reported APOs showed less incidence in our data than in previous research. For example, for preterm delivery, our results identified an incidence of 2.95%; however, 6.5% has been reported for Hong Kong [44] and 4.16% for Guangdong Province [10]. Previous research has shown that self-reported data may show considerable misclassification of new occurrences of diseases, including high specificity. Henceforth, self-reported incidence of disease would result in rarely giving false-positive results with low sensitivity, meaning considerable false negativity [45]. Though all doctors seemed well trained and skillful, this drawback of self-reporting was inevitable, like other previous research. Data quality related to self-reported APOs could underestimate our present research’s incidence, which might explain the differences. The target of our research was restricted to depicting the spatial-temporal distribution to reduce this negative influence supposing this false negative was common and consistent at the county level or in tempo. The relatively lower incidence of APOs is also partly explained by spatial heterogeneity. A cross-sectional study has reported a relatively higher incidence of preterm delivery of 4.66% for China compared with our results. However, the incidence for Henan (3.93%) is lower than in the other two provinces: Sichuan (4.78) and Anhui (5.23), in this previous research [46].

There are some strengths in our study. It was a population-based surveillance study for APOs in rural areas of Henan Province, which also has a large population. It is one of the few to describe the overall situation of APOs in rural Henan Province, China, providing clues for the epidemiological distribution of APOs in rural China and future analysis of the causal effect of demographic factors. The population design and high population coverage (over 80%) minimized the selection bias. However, the potential limitations should also be acknowledged. First, due to privacy protection, the smallest spatial unit of samples was only at the county level; therefore, the spatial variation inside the counties could not be distributed. Second, we only analyzed the spatial-temporal distribution of APOs but did not consider environmental factors in this study. Further research into the environmental factors of APOs in Henan could be conducted based on these spatial-temporal patterns.

## 5. Conclusions

This study demonstrates the decreasing trends, seasonal fluctuation, and clustered patterns of APOs across space and over time in Henan Province of China. Future studies will build upon the findings of this study, investigating how environmental factors could account for the variation in distribution patterns of congenital disabilities.

## Figures and Tables

**Table 1:** Characteristics of the participants.

Characteristic	Adverse Pregnancy Outcomes		*p*
	Yes (*n* = 160,383)	No (*n* = 1,154,944)	
Maternal age			<0.001 *
(20, 25)	45,306 (11.80%)	338,611 (88.20%)	
(25, 30)	83,920 (12.23%)	602,031 (87.77%)	
(30, 35)	21,650 (12.42%)	152,673 (87.58%)	
(35, 40)	7276 (13.12%)	48,170 (86.88%)	
(40, 45)	1888 (13.81%)	11,784 (86.19%)	
(45, 50)	343 (17.00%)	1675 (83.00%)	
Education level completed			<0.001 *
Missing	4989 (15.25%)	27,734 (84.75%)	
College or higher	17,770 (14.50%)	104,798 (85.50%)	
Senior high school	1520 (12.68%)	10,471 (87.32%)	
Junior high school	26,395 (12.81%)	179,693 (87.19%)	
Primary school or below	109,709 (11.65%)	832,248 (88.35%)	
Maternal occupation			<0.001
Farmer	138,965 (11.89%)	1,029,831 (88.11%)	
Other	21,418 (14.62%)	125,113 (85.38%)	
Maternal ethnicity			<0.001
Han	156,389 (12.15%)	1,130,794 (87.85%)	
Other	3994 (14.19%)	24,150 (85.81%)	
Maternal BMI			<0.001 *
<18.5	15,221 (12.70%)	104,627 (87.30%)	
18.5–23.9	116,835 (12.01%)	856,197 (87.99%)	
24.0–27.9	22,540 (12.67%)	155,357 (87.33%)	
≥28.0	5564 (13.01%)	37,194 (86.99%)	
Missing	223 (12.44%)	1569 (87.56%)	
Season of conception			<0.001
Spring	49,473 (12.66%)	341,325 (87.34%)	
Summer	40,332 (12.34%)	286,477 (87.66%)	
Autumn	32,561 (11.16%)	259,263 (88.84%)	
Winter	38,017 (12.43%)	267,879 (87.57%)	
Maternal pre-gestational smoking			<0.001
Missing	616 (0.38%)	4093 (0.35%)	
None	158,573 (98.87%)	1,142,311 (98.91%)	
Yes	1184 (0.74%)	8492 (0.74%)	
Maternal pre-gestational drinking			<0.001
Missing	616 (13.08%)	4093 (86.92%)	
No	158,573 (12.19%)	1,142,311 (87.81%)	
Occasionally	1184 (12.24%)	8492 (87.76%)	
Often	10 (17.24%)	48 (82.76%)	
Paternal pre-gestational drinking			<0.001
Missing	1,838 (14.30%)	11,016 (85.70%)	
No	119,913 (11.93%)	884,923 (88.07%)	
Occasionally	37,929 (12.94%)	255,161 (87.06%)	
Often	703 (15.46%)	3844 (84.54%)	
Paternal pre-gestational smoking			<0.001
Missing	1710 (14.10%)	10,416 (85.90%)	
None	123,761 (11.99%)	908,074 (88.01%)	
Yes	34,912 (12.87%)	236,454 (87.13%)	
Region ^1^			<0.001
East	49,228 (13.15%)	325,085 (86.85%)	
West	12,038 (9.46%)	115,203 (90.54%)	
Middle	23,827 (12.69%)	163,970 (87.31%)	
South	46,429 (11.36%)	362,457 (88.65%)	
North	28,861 (13.30%)	188,229 (86.71%)	

^1^ East (Kaifeng City, Shangqiu City, Zhoukou City), west (Luoyang City and Sanmenxia City), central (Zhengzhou City, Pingdingshan City, Xuchang City, Luohe City), south (Nanyang City, Zhumadian City, Xinyang City) and north (Anyang City, Xinxiang City, Jiaozuo City, Puyang City, Hebi City and Jiyuan City); * Kolmogorov-Smirnov Tests were used to test the significance of the cumulative distribution functions between subgroups.

**Table 2:** The seasonal incidence rates of APOs in Henan Province from 2013 to 2016.

Pregnancy Outcomes	Autumn		Spring		Summer		Winter		*p* Values
	*n*	%	*n*	%	*n*	%	*n*	%	
PreD	7431	2.55 *	12,421	3.18 ^Δ^	9626	2.95	9292	3.04	<0.001
LBWI	1952	0.67	2717	0.70	2123	0.65 *	2173	0.71 ^Δ^	0.015
MedA	780	0.27	1149	0.29	933	0.29	829	0.27	0.13
TIL	270	0.09	352	0.09	320	0.1	304	0.1	0.553
SponD	2828	0.97 *	4024	1.03	3819	1.17 ^Δ^	3568	1.17 ^Δ^	<0.001
StiB	195	0.07	269	0.07	222	0.07	231	0.08	0.568
ProD	3911	1.34	5276	1.35	4940	1.51 ^Δ^	4027	1.32 *	<0.001
MF	17,117	5.87 *	26,268	6.72 ^Δ^	20,602	6.3	19,905	6.51	<0.001
APOs	32,563	11.16 *	49,474	12.66 ^Δ^	40,332	12.34	38,018	12.43	<0.001

SponA: spontaneous abortion, PreD: preterm delivery, LBWI: low birth weight infant, MacF: macrosomia fetus, ProD: prolonged delivery, StiB: stillbirth, MedA: medical abortion, tInL: therapeutic induce of labour, APOs: adverse pregnancy outcomes; *: the highest seasonal incidence rate for each APO separately and all APOs; ^Δ^: the lowest seasonal incidence rate for each APO separately and all APOs.

## Data Availability

The data are available from the corresponding author by reasonable request.
